# *Clostridium manihotivorum* sp. nov., a novel mesophilic anaerobic bacterium that produces cassava pulp-degrading enzymes

**DOI:** 10.7717/peerj.10343

**Published:** 2020-11-16

**Authors:** Pattsarun Cheawchanlertfa, Sawannee Sutheeworapong, Piroon Jenjaroenpun, Thidathip Wongsurawat, Intawat Nookaew, Supapon Cheevadhanarak, Akihiko Kosugi, Patthra Pason, Rattiya Waeonukul, Khanok Ratanakhanokchai, Chakrit Tachaapaikoon

**Affiliations:** 1 School of Bioresources and Technology, King Mongkut’s University of Technology Thonburi, Bangkok, Thailand; 2 Pilot Plant Development and Training Institute, King Mongkut’s University of Technology Thonburi, Bangkok, Thailand; 3 Division of Bioinformatics and Data Management for Research, Department of Research and Development, Faculty of Medicine, Siriraj Hospital, Mahidol University, Bangkok, Thailand; 4 Department of Biomedical Informatics, College of Medicine, University of Arkansas for Medical Sciences, Little Rock, AR, USA; 5 Department of Physiology and Biophysics, College of Medicine, University of Arkansas for Medical Sciences, Little Rock, AR, USA; 6 Biological Resources and Post-harvest Division, Japan International Research Center for Agricultural Sciences, Ibaraki, Japan

**Keywords:** Cassava pulp-degrading enzymes, *Clostridium* species, Complete genome sequence, Illumina, Mesophilic anaerobic bacterium, Oxford nanopore technology

## Abstract

**Background:**

Cassava pulp is a promising starch-based biomasses, which consists of residual starch granules entrapped in plant cell wall containing non-starch polysaccharides, cellulose and hemicellulose. Strain CT4^T^, a novel mesophilic anaerobic bacterium isolated from soil collected from a cassava pulp landfill, has a strong ability to degrade polysaccharides in cassava pulp. This study explored a rarely described species within the genus *Clostridium* that possessed a group of cassava pulp-degrading enzymes.

**Methods:**

A novel mesophilic anaerobic bacterium, the strain CT4^T^, was identified based on phylogenetic, genomic, phenotypic and chemotaxonomic analysis. The complete genome of the strain CT4^T^ was obtained following whole-genome sequencing, assembly and annotation using both Illumina and Oxford Nanopore Technology (ONT) platforms.

**Results:**

Analysis based on the 16S rRNA gene sequence indicated that strain CT4^T^ is a species of genus *Clostridium*. Analysis of the whole-genome average amino acid identity (AAI) of strain CT4^T^ and the other 665 closely related species of the genus *Clostridium* revealed a separated strain CT4^T^ from the others. The results revealed that the genome consisted of a 6.3 Mb circular chromosome with 5,664 protein-coding sequences. Genome analysis result of strain CT4^T^ revealed that it contained a set of genes encoding amylolytic-, hemicellulolytic-, cellulolytic- and pectinolytic enzymes. A comparative genomic analysis of strain CT4^T^ with closely related species with available genomic information, *C. amylolyticum* SW408^T^, showed that strain CT4^T^ contained more genes encoding cassava pulp-degrading enzymes, which comprised a complex mixture of amylolytic-, hemicellulolytic-, cellulolytic- and pectinolytic enzymes. This work presents the potential for saccharification of strain CT4^T^ in the utilization of cassava pulp. Based on phylogenetic, genomic, phenotypic and chemotaxonomic data, we propose a novel species for which the name *Clostridium manihotivorum* sp. nov. is suggested, with the type strain CT4^T^ (= TBRC 11758^T^ = NBRC 114534^T^).

## Introduction

The bio-based economy is an emerging sector with a notable potential for economic growth and with promising business opportunities. It is generally defined as the sustainable exploitation and management of renewable natural resources for producing bio-based products. Recently, biorefineries utilize lignocellulosic and other organic raw materials to generate a spectrum of bio-based products such as biofuels, biochemicals and other high value-added products get attention (FitzPatrick et al., 2010). Biomass feedstocks are grouped into two categories, carbohydrate-rich and oleaginous (Melero, Iglesias & Garcia, 2012). Carbohydrate-rich feedstocks contain starch and non-starch polysaccharides (NSP). Industrial starch-rich by-products such as cassava pulp, wheat bran, rice bran, sago pith residues and brewery-spent grains are available in enormous quantities and vary in terms of starch and NSP, hemicellulose and cellulose components (Cripwell et al., 2015). These materials are potential feedstocks for bio-based production, however, they have first to undergo a pretreatment process for the enhanced production of biofuels, organic acids and other valuable biochemicals (Cripwell et al., 2015; Zhang et al., 2016). The starch granules in the starch-rich by-products are entrapped tightly in the secondary cell wall structure by cellulose, hemicellulose and lignin, thus, the starch cannot be easily released for further conversion (Apiwatanapiwat et al., 2016). Moreover, the costs associated with the pretreatment process, such as the energy, equipment and wastewater treatment costs, have resulted in the slow adoption of the technology.

Thailand is a major cassava producer for the domestic and global markets. Cassava starch factories in Thailand generate approximately 1.5–2.0 million tons of waste cassava pulp annually (Norrapoke et al., 2018). Most of the cassava pulp ends up in landfills, resulting in environmental pollution. The pulp spoils rapidly in the humid, warm tropical environment, and under anaerobic conditions generates methane, thus contributing to global warming and leaching of the soil, entering water sources and creating a nuisance to the air quality near the cassava starch factories that consequently affects human health. The utilization rather than discarding of cassava pulp will, therefore, reduce the negative impact on environmental and human health. On a dry weight basis, cassava pulp is mainly composed of starch (50–60%, w/w) with 15–27% (w/w) cellulose and hemicelluloses contents, pectin (7.0–7.3%, w/w), and lignin (3.4–4.6%, w/w) (Djuma’ali et al., 2012; Vaithanomsat et al., 2013). In general, the saccharification of cassava pulp to fermentable sugars used in the production of high value-added products requires the action of enzymes belonging to glycoside hydrolases (GHs), which hydrolyze the glycosidic bonds of starch, cellulose and hemicellulose contained in lignocellulose (Naumoff, 2011; Linares-Pastén, Andersson & Karlsson, 2014). Thus, the hydrolysis of cassava pulp by enzymatic saccharification requires the interaction of a set of carbohydrate-active enzymes containing amylolytic, cellulolytic and hemicellulolytic activities (Rattanachomsri et al., 2009).

Mesophilic anaerobic Clostridia have been reported to produce enzymes that have a high potential to hydrolyze biomass feedstocks (Doi & Kosugi, 2004). However, there are different dominant groups of enzymes to degrade starch and NSP. Although *Clostridium polyendosporum* PS-1^T^ (Duda et al., 1987) and *C. amylolyticum* SW408^T^ (Song & Dong, 2008) have been reported to utilize starch as their carbon source for growth, until now no further studies on the amylolytic enzyme properties of these two microorganisms have been elucidated. So far, the genome of *C. amylolyticum* SW408^T^ has been sequenced by the Joint Genome Institute (JGI) as part of the Community Science Program in 2016, revealing a total of 27 genes coded for amylolytic-, hemicellulolytic- and cellulolytic-enzymes that mainly consisted of genes encoding for an amylolytic enzyme, with very low hemicellulolytic- and cellulolytic-enzymes, while the *C. polyendosporum* PS-1^T^ genome is not available and has not been reported in the database. In comparison, mesophilic anaerobic Clostridia, such as *C. cellulovorans* 743B^T^ (Sleat, Mah & Robinson, 1984; Tamaru et al., 2010), *C. cellulolyticum* ATCC 35319^T^ (Desvaux, 2005), *C. josui* JCM 17888^T^ (Sakka et al., 2010), *C. acetobutylicum* ATCC 824^T^ (Sabathé, Soucaille & Bélaïch, 2002) and *C. bornimense* M2/40^T^ (Tomazetto et al., 2016) produce highly active cellulolytic enzymes, but provide very low amylolytic- and hemicellulolytic-enzyme activities. In contrast, *Clostridium* sp. strain MF28 was reported as producing a highly hemicellulolytic enzyme with an efficient capability to degrade hemicelluloses and raw plant biomass, but which expressed a low level of amylolytic- and cellulolytic-enzymes (Li & He, 2016). To date, there has been no report of any mesophilic anaerobic *Clostridium* capable of producing an array of amylolytic-, hemicellulolytic- and cellulolytic-enzymes which can degrade cassava pulp efficiently. Therefore, only the enzyme systems from microorganism that is infrequently isolated, especially those from mesophilic anaerobic bacteria, possess the ability to degrade starch-based biomass and may, therefore, provide increased opportunities for industrial applications. These bacteria have always predominantly produced a wide range of pH and temperature tolerant enzymes (Himmel et al., 2010). They are very suitable to be used on starch liquefaction and saccharification processes and can save energy, reduce expensive heating steps and reduce adverse chemical reactions at high temperatures. This observation has encouraged us to look for a new mesophilic anaerobic bacterium that can produce an array of amylolytic-, hemicellulolytic- and cellulolytic-enzymes that will function synergistically and cooperatively to degrade cassava pulp, as well as hydrolyze the recalcitrant cell wall structure of the pulp.

The usual parameters used to delineate and describe new bacterial species include 16S ribosomal RNA gene sequence-based identity and phylogeny (Tindall et al., 2010), genomic G + C content diversity and DNA-DNA hybridization (DDH) (Wayne et al., 1987). However, there are some limitations to these parameters, notably because the cutoff values vary dramatically between genera and species. The introduction of high-throughput sequencing techniques has made genomic data available for many bacterial species, and to date, the availability of low-cost, high-performance sequencing continues to expand the diversity of research and applications on a genome-scale. Advances in the next generation of sequencing technologies, e.g., Illumina (Bentley et al., 2008) and Oxford Nanopore Technology (ONT) platforms (Clarke et al., 2009) have been applied to sequencing full-length genetic information of many organisms, by generating short- and long-read sequence data that enables the accurate identification of species-level taxonomy and allows for the de novo assembly of complete genomes. The combination of genomic and phenotypic information will allow a faster and more reliable classification of new isolates of microorganisms (Chun & Rainey, 2014).

In this study, we isolated a novel mesophilic anaerobic bacterium, *Clostridium manihotivorum* CT4^T^ from the soil of a cassava pulp landfill. The isolated strain demonstrated an efficient degradation of cassava pulp, a by-product of the cassava starch industry. The phenotypic and biochemical characteristics of the isolated strain were reported. To better understand the genetic basis for the cassava pulp degradation by strain CT4^T^, its genome was entirely sequenced using Illumina and ONT platforms. The genome analysis of strain CT4^T^ identified a set of genes encoding amylolytic-, hemicellulolytic- and cellulolytic-enzymes critical to its ability to degrade cassava pulp, which is rarely found in *Clostridium* species.

## Materials and Methods

### Preparation of samples and basal medium

Samples of cassava pulp and soil beneath the waste heap were obtained from a starch factory landfill in Chonburi Province, Thailand. The pulp was ground by an ultra centrifugal mill ZM-100 and sieved through a 0.5 mm mesh screen (Retsch, Haan, Germany). The pulp was washed several times with distilled water to remove the remaining sugar and other dirt, oven-dried at 50 °C until at a constant weight and then stored in plastic bags at 4 °C for further experiments.

The basal medium (BM7; pH 7.0) was composed of (per liter) 1.5 g KH_2_PO_4_, 2.9 g K_2_HPO_4_, 2.1 g urea, 4.5 g yeast extract, 0.5 g cysteine-HCl, 0.001 g resazurin and 200 µL mineral solution (25.0 g/L MgCl_2_.6H_2_O, 37.5 g/L CaCl_2_.2H_2_O and 0.3 g/L FeSO_4_.6H_2_O). The BM7 was anaerobically prepared in bottles sealed with butyl rubber stoppers, under an atmosphere of high-purity N_2_, and sterilized by autoclaving at 121 °C for 15 min.

### Screening and isolation of cassava pulp-degrading strains

The enrichment and isolation were performed under anaerobic conditions. Approximately 1 g of the soil sample was transferred into Hungate tubes containing 15 mL BM7 (pH 7.0) and 1% (w/v) cassava pulp. After inoculation, each test tube was flushed with N_2_ and incubated at 37 °C. The culture that showed the highest degradation of pulp, as visually indicated by the remaining cassava pulp (approximately ≤ 50% residue dry weight), was selected and serially diluted into agar-cassava pulp medium that had been preliminarily melted and cooled to 55 °C. The cultures were then subjected to the roll-tube technique for isolating obligate anaerobes (Hungate, 1969), after which solidified samples were incubated at 37 °C. Single colonies were isolated with sterile needles and inoculated into BM7 broth containing cassava pulp. Afterward, the cultures were incubated to study their ability to degrade the cassava pulp. Pure cultures were obtained following repeated sub-culturing (ten times) in BM7 containing cassava pulp.

The composition of cassava pulp and residual cassava pulp digested by *C. manihotivorum* CT4^T^, *C. polyendosporum* PS-1^T^ and *C. amylolyticum* SW408^T^ were analyzed following the National Renewable Energy Laboratory (NREL) protocol (Sluiter et al., 2008).

### 16S rRNA gene sequencing

Genomic DNA for 16S rRNA gene sequencing was prepared by phenol-chloroform extraction. The 16S rRNA gene was amplified by PCR using the following primers: 8F (5′–AGAGTTTGATCCTGGCTCAG–3′) and 1492R (5′–GGTTACCTTGTTACGACTT–3′). The PCR reaction conditions were as follows: 94 °C for 3 min, 35 cycles at 94 °C for 30 s, 55 °C for 40 s and 72 °C for 2 min, with a final extension time of 5 min at 72 °C. The amplified fragment was ligated into the pGEM-T Easy vector (Promega, Madison, WI, USA), and the recombinant plasmid was sequenced using T7 and SP6 primers. A sequence similarity search was performed using the BLAST program (http://blast.ncbi.nlm.nih.gov/Blast.cgi). A phylogenetic tree was generated by the neighbor-joining method with 1,000 bootstrap replications, employing the MEGA version 6.0 (Tamura et al., 2013).

### Physiological and biochemical analysis

Gram staining of strain CT4^T^ was conducted using the conventional methodology and confirmed using the KOH test (Powers, 1995). Endospore staining was examined by Schaeffer–Fulton’s method (Schaeffer & Fulton, 1933). Cell morphology was observed by scanning electron microscope (SEM; model SU800 Hitachi, Japan). Substrate utilization was tested by growing the strain in BM7 containing 0.5% (w/v) of the following substrates: D-glucose, D-galactose, D-arabinose, D-rhamnose, D-mannose, D-xylose, D-fructose, D-trehalose, D-raffinose, lactose, sucrose, maltose, cellobiose, mannitol, soluble starch from potato (ACS reagent), birchwood xylan, cellulose powder (Type 20) and Avicel^®^ (PH-101); these chemicals were purchased from Sigma-Aldrich, Saint Louis, MO, USA. Raw cassava starch and cassava pulp were obtained from a starch factory landfill in Chonburi Province, Thailand. After 5 days of incubation, cell growth was assessed by determining the optical density at 600 nm. The fermentation products in the supernatant extracted from the glucose-grown culture were determined by gas chromatography equipped with a flame-ionization detector and a Carbopack B-DA/4% Carbowax 20M column (GC-14A; Shimadzu, Japan). The column, injector and detector temperatures were 170, 230 and 230 °C, respectively. Other biochemical tests were conducted according to the methods of Holdeman, Cato & Moore (1977) and Summanen et al. (1993). The isomers of diaminopimelic acid (DAP) in the cell wall were determined as described by Komagata and Suzuki (Komagata & Suzuki, 1988). Cellular fatty acids were extracted, methylated and analyzed using the standard microbial identification system (MIDI) protocol (Sherlock microbial identification system, version 6.1) while the fatty acids were identified using the TSBA6 database of the microbial identification system (Sasser, 1990). The polar lipids were analyzed from freeze-dried cells by two-dimensional TLC, as described by Minnikin et al. (1984). Appropriate detection reagents were used to visualize the spots: phosphomolybdic acid reagent 5% (w/v) solution in ethanol (Sigma-Aldrich, Saint Louis, MO, USA) was used to detect total polar lipids; ninhydrin reagent (0.2% solution; Sigma-Aldrich) was used to detect amino lipids; Dittmer and Lester reagent (molybdenum blue reagent, 1.3%; Sigma-Aldrich) was used to detect phospholipids and Dragendorff’s reagent (Sigma-Aldrich) was used to detect phosphatidylcholine.

### Cultivation and enzyme production

Strain CT4^T^ was anaerobically cultivated in 1,000 mL of BM7 containing 1% (w/v) cassava pulp for 3 days at 37 °C, pH 7.0 under static conditions in an anaerobic chamber (Bactron II, USA). The culture supernatant was collected by centrifugation at 10,000× *g* for 10 min at 4 °C and subsequently concentrated using a hollow fiber cartridge with a 10-kDa-cutoff membrane (GE Healthcare, USA). The retentate (50-times more concentrated) was then used as the crude enzyme.

### Enzyme assays and protein determination

All enzyme assays were performed in 50 mM sodium phosphate buffer (pH 7.0) and incubated at 55 °C for 10 min. The enzymatic activities on 1% (w/v) cassava pulp, soluble starch, pullulan, birchwood xylan, cellulose powder (Type 20) and pectin from citrus peel were assayed by determining the amount of reducing sugars liberated by the Somogyi–Nelson method (Nelson, 1944). One unit (U) of enzyme activity was defined as the amount of enzyme that released 1 µmol of reducing sugar in 1 min under assay conditions. The protein concentration was determined by the Lowry method (Lowry et al., 1951) using bovine serum albumin as a standard.

### Library preparation and genome sequencing

The genomic DNA was extracted from the cultures using a blood and cell culture DNA midi kit (Qiagen, Germany) according to the manufacturer’s instructions. Strain CT4^T^ was sequenced using two sequencing platforms: Illumina (Hiseq2500) and ONT (MinION). Illumina sequencing paired-end DNA libraries were prepared following the Illumina DNA manufacturer’s instructions (NEBNext^^®^^ Ultra™ DNA library prep kit). The size-selected, adaptor-ligated DNA fragments were PCR-amplified using the following protocol: polymerase activation (98 °C for 30 s), followed by 10 cycles (denaturation at 98 °C for 10 s, annealing at 65 °C for 75 s and extension at 65 °C for 75 s) with a final 5 min extension at 65 °C. The DNA libraries were purified by magnetic beads, and their size distribution was checked using Agilent Bioanalyzer DNA high sensitivity chip assay. The DNA fragments were sequenced using the Illumina Hiseq2500 with 2 × 150 bp paired-end protocol (Illumina, Inc., California, USA).

The ONT library preparation and bioinformatics analysis were performed according to Jenjaroenpun et al. (2018). In brief, a total amount of 600 ng genomic DNA was used as input for a rapid sequencing kit (SQK-RAD002) to generate the DNA sequencing library. The library was then loaded onto a flow cell version FLO-MIN106 on a MinION Mk1B (released in 2014 through the MinION Access Program) (Oxford, UK) to perform DNA sequencing for 48 h. Base-calling was performed using the local-based software, Albacore version 1.2.3 (ONT, USA).

### Genome assembly and annotation

The high-quality ONT reads (average quality score of >7) were first assembled using combination Minimap2 (Li, 2018) and Miniasm (Li, 2016), resulting in a circular draft genome. The draft genome was polished using Racon (Vaser et al., 2017) and Nanopolish (Loman, Quick & Simpson, 2015), based on the consensus pileup of high-quality ONT reads and additionally using Pilon (Walker et al., 2014), based on short Illumina reads.

Genome annotation was conducted with a Prokka annotation pipeline (Seemann, 2014). The rRNA and tRNA genes were identified with RNAmmer (Lagesen et al., 2007) and Aragorn software (Laslett & Canback, 2004), respectively. Functional classification of protein-coding genes in *Clostridium* sp. strain CT4^T^ was done by assigning Cluster of Orthologous Group (COG) codes to each gene using eggnog-Mapper (Huerta-Cepas et al., 2017) and eggNOG version 4.5 (Huerta-Cepas et al., 2016).

### Average amino acid identity analysis

The first analysis comprised pairwise comparisons of AAIs (Konstantinidis & Tiedje, 2005) of the 665 genomes belonging to the Clostridia class (Cabal et al., 2018). For each pair of genomes, the average AAI was then calculated based on the identities of all conserved reciprocal best matches, a calculation that was not always symmetrical. In such cases, the average of the two AAI values was assigned to each pair of genomes. The AAI tree was built with BIONJ (Gascuel, 1997) to find dissimilarities of AAI values (100% minus AAI).

### Comparison of glycoside hydrolase producing genes in strain CT4^T^ with related species

Strain CT4^T^ (GenBank accession number CP025746) was compared with the closely related *C. amylolyticum* SW408^T^ (NZ_FQZO00000000; NCBI) that had available genomic information in the NCBI database, using OrthoMCL (Chen et al., 2006) to characterize their specific genetic features and identify overlaps among orthologous clusters. The protein sequences were grouped into gene families encoding amylolytic-, hemicellulolytic- and cellulolytic-enzymes, using the criteria: *E*-value <1E-5 and sequence identity >50%. The genomic information of *C. polyendosporum* PS-1^T^ was not reported in the NCBI database, therefore, the strain PS-1^T^ was excluded from genome comparison.

## Results

### Isolation and identification of cassava pulp-degrading bacterium

**Figure 1:**
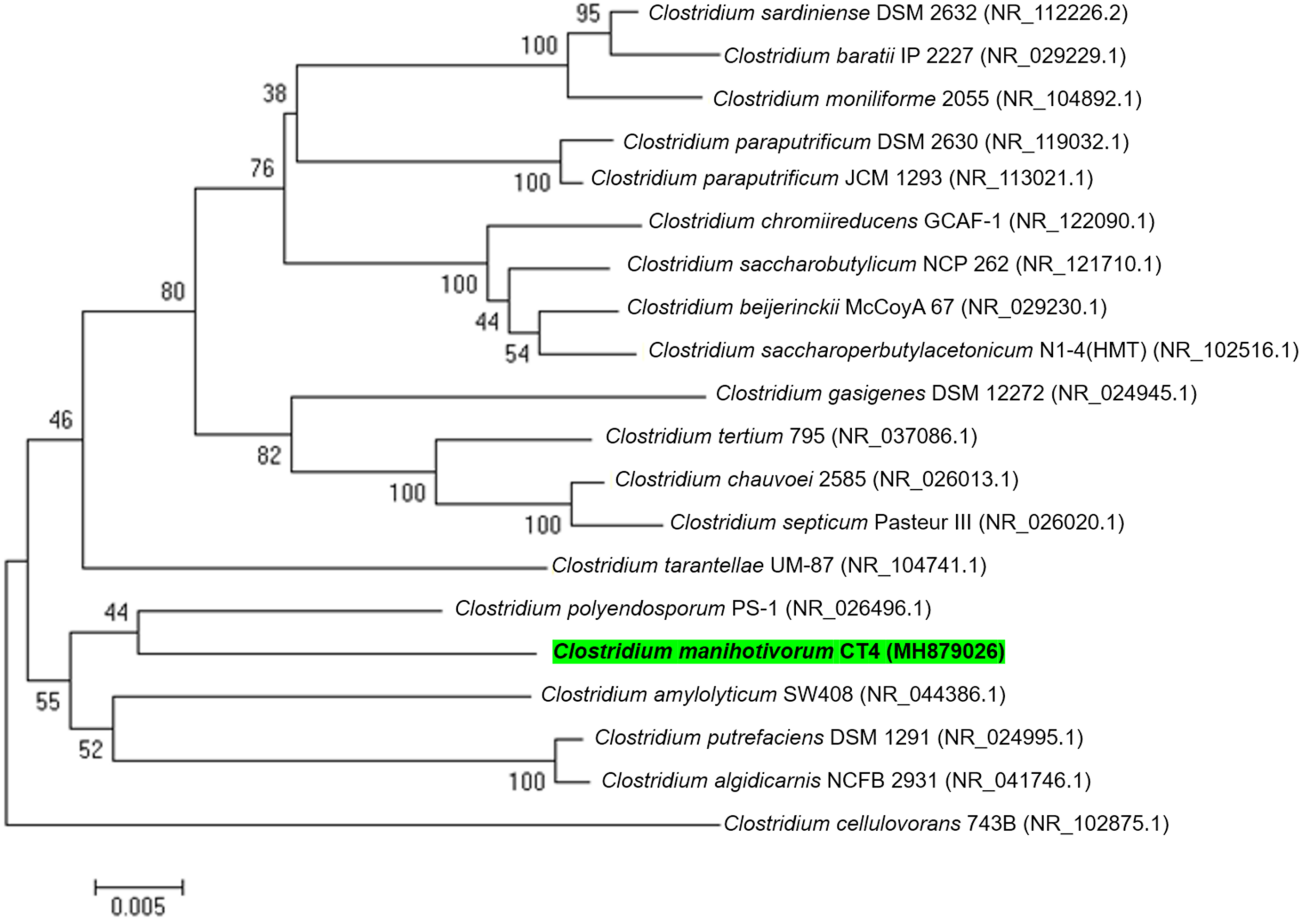
A phylogenetic tree was constructed from 16S rRNA gene sequences by the neighbor-joining method that showed the relatedness of *C. manihotivorum* CT4^T^ with other members of the genus *Clostridium*.

In total, 15 individual colonies were isolated by the roll-tube technique and were subcultured 10 times in BM7 separately, utilizing cassava pulp as carbon sources. Visualization of the roll-tube appearance revealed that isolate CT4^T^ performed best in relation to cassava pulp degradation. Moreover, approximately 60.8% (w/v) removal of dry weight was detected when cultured in BM7 broth. The isolated CT4^T^ thoroughly utilized the starch contains in cassava pulp after 5 days of culturing. The result showed that the isolated CT4^T^ could remove 99.0% starch in cassava pulp, while cellulose and hemicellulose contents were removed by 42.2% and 39.2%, respectively, when compared with the control. Besides, this strain removed starch and non-starch polysaccharide in cassava pulp better than the related species, strain PS-1^T^ and SW408^T^ (Fig. S2). Thus, it was consequently selected for further analysis. Prior to genome sequencing, the 16S rRNA gene sequence of strain CT4^T^ (accession number MH879026) was compared with the nucleotide sequences in NCBI. The analysis revealed that strain CT4^T^ shared 95% sequence identity with *Anaerobacter polyendosporus* PS-1^T^, now reclassified as *Clostridium polyendosporum* comb. nov. (Duda et al., 1987; Stackebrandt et al., 1999) and 94% sequence identity with *C. amylolyticum* SW408^T^ (Song & Dong, 2008), *Clostridium putrefaciens* DSM 1291^T^ (Sturges & Drake, 1927), and *Clostridium algidicarnis* NCFB 2931^T^ (Lawson et al., 1994). Phylogenetic analysis based on 16S rRNA gene sequences and neighbor-joining method indicated that strain CT4^T^ belongs to the genus *Clostridium* (Fig. 1). Therefore, isolated CT4^T^ was classified as *Clostridium* sp. CT4^T^

### Physiological and biochemical characteristics of strain CT4^T^

**Figure 2:**
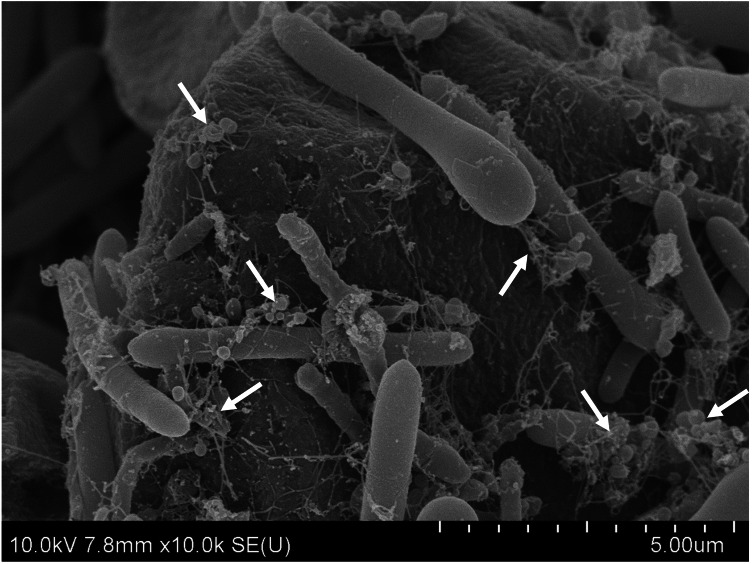
The SEM of *C. manihotivorum* CT4^T^ grown on basal medium with cassava pulp as the sole carbon source.

The SEM image revealed that cells of strain CT4^T^ were rod-shaped, and surrounded by a polysaccharide capsule (Fig. 2). Strain CT4^T^ was Gram-positive, single endospore-forming, non-motile and non-flagellate (Table 1). To understand the optimal growth conditions, strain CT4^T^ was cultivated under different pH (pH 4.0–11.0) and temperature (25−50 °C) conditions. Strain CT4^T^ could grow at a wide range of temperatures (25−45 °C) and pH (5.5–7.5) in BM7 medium containing 1% (w/v) cassava pulp. The optimum growth of strain CT4^T^ was found at 37 °C and pH 7.0. Moreover, strain CT4^T^ used a wide range of carbon sources, including D-glucose, D-xylose, D-galactose, D-fructose, D-mannose, D-arabinose, D-rhamnose, D-trehalose, D-raffinose, sucrose, lactose, maltose, mannitol, cellobiose, soluble starch, xylan, cellulose and Avicel^^®^^. The main metabolic products of strain CT4^T^, ranked based on quantity, were acetate, butyrate, ethanol and propionate. Butanol was not observed during growth, whereas it was produced in the closest relative, *C. polyendosporum* PS-1^T^ (Table 1). In contrast, *C. putrefaciens* isolated from spoiled ham (Sturges & Drake, 1927), and *C. algidicarnis* isolated from vacuum-packed refrigerated pork (Lawson et al., 1994) cannot hydrolyze starch despite their relatedness to strain CT4^T^ (Table 1). Strain CT4^T^ presented *LL*-diaminopimelic acid (*LL*-DAP) in their cell wall, whereas most members in the genus *Clostridium* contains meso-diaminopimelic acid. Thus, the strain CT4^T^ was different from the other related strains, except *C. putrefaciens* that have the same with strain CT4^T^. The cellular fatty acid profiles of strain CT4^T^ are listed in Table S1. The major fatty acids detected from strain CT4^T^ were C _16:0_ (37.4%), C _14:0_ (15.0%), anteiso-C _15:0_ (5.5%), summed feature 1 (C _13:0_-3OH and/or C _15:1_isoH; 4.5%), C _19:0_cyclo *ω*8c (4.2%) and C _17:0_2-OH (4.0%). In terms of their polar lipid profiles, strain CT4^T^ contained phosphatidylethanolamine (PE) and phosphatidylglycerol (PG) as the major polar lipids, while phosphatidylcholine (PC) was found as minor polar lipid. Additionally, three unidentified phospholipids (PL1–PL3) and three unidentified amino lipids (AL1–AL3) were also detected (Fig. S1).

**Table 1:** The phenotypic characteristics of *C. manihotivorum* CT4^T^ and its close phylogenetic neighbors.

**Characteristic**	**1**	**2**	**3**	**4**	**5**
Isolation source	Decomposed cassava pulp soil	Meadow soil	H_2_-producing upflow anaerobic sludge blanket reactor	Ham	Vacuum-packed refrigerated pork
Cell morphology	Rod	Rod	Rod	Rod	Rod
Cell length/width (µm)	4−8/0.5−1.5	4−8/1.5−3.0	2.0−7.5/0.5−0.7	3−15/0.5−0.7	2−5/0.5−1.0
Gram-stain	+	+	+	+	+
Endospores formed (amount/cell)	+ (1)	+ (up to 7)	+ (1)	+ (1)	+ (1)
Motility	−	−	+	−	−
Flagella	−	NR	+	NR	NR
Temperature range/ optimum (°C)	25−45/37	15−45/25−35	24−45/37	20−25	25−30
pH range/optimum	5.5−7.5/7.0	5.5−8.5/6.5 −7.5	4.0−9.0/7.0	6.0−9.0/8.0	NR
Starch degradation	+	+	+	−	−
Fermentation products^a^	A, B, E, P, CO_2_, H_2_	A, B, E, L, CO_2_, H_2_, b	A, E, CO_2_, H_2_	NR	A, B
G + C (mol%)	32	29	33	NR	NR
Reference	This study	Duda et al. (1987)	Song & Dong (2008)	Sturges & Drake (1927)	Lawson et al. (1994)

**Notes.**

Strains: 1, strain CT4^T^; 2, *Clostridium polyendosporum* PS-1^T^; 3, *Clostridium amylolyticum* SW408^T^; 4, *Clostridium putrefaciens* DSM 1291^T^; 5, *Clostridium algidicarnis* NCFB 2931^T^.

a Fermentation products from 1% (w/v) glucose: A, acetate; B, butyrate; E, ethanol; L, lactate; P, propionate; b, butanol.

Notes: −, negative; +, positive; NR, no reported.

Based on the 16S rRNA gene sequence similarity, physiological attributes and biochemical properties, strain CT4^T^ was considered to be a novel species of the genus *Clostridium*. Thus, the strain CT4^T^ was introduced in the namely *Clostridium manihotivorum* CT4^T^, which can degrade cassava pulp. The meaning of “*manihotivorum*” is devouring cassava. This bacterium was deposited as a type strain in the Thailand Bioresource Research Center (TBRC), and NITE Biological Resource Center (NBRC), Japan under accession numbers TBRC 11758^T^ and NBRC 114534^T^, respectively.

### Characterizations of amylolytic-, hemicellulolytic- and cellulolytic-enzymes of *C. manihotivorum* CT4^T^

In this study, a *C. manihotivorum* CT4^T^ was discovered to degrade cassava pulp, which was able to produce the cassava pulp degrading enzymes, including amylolytic-, hemicellulolytic- and cellulolytic-enzymes. In order to characterize the properties of the crude enzyme from strain CT4^T^, the isolate was cultivated in BM7 medium containing 1% (w/v) cassava pulp at pH 7.0, 37 °C. Afterwards, the culture supernatant was harvested at the early stationary phase (3 days) and concentrated by ultrafiltration technique. The crude enzyme gave the highest activity on cassava pulp (1,901.1 U/g protein), which was 1.56-fold higher than that obtained from soluble starch (1,212.7 U/g protein). In addition, a pullulanase activity of 27.5 U/g protein was detected (Table 2). *C. manihotivorum* CT4^T^ was also able to produce xylanase (43.5 U/g protein), cellulase (32.0 U/g protein) and pectinase (42.4 U/g protein) as shown in Table 2, which are involved in the degradation of xylan, cellulose and pectin contained in the cell wall structure of cassava pulp, respectively.

**Table 2:** The enzymatic activities of the crude enzyme from *C. manihotivorum* CT4^**T**^.

**Enzyme**	**Specific activities(U/g protein)**
Cassava pulp-degrading enzyme^a^	1,901.1 ± 57.4
Amylase^b^	1,212.7 ± 22.8
Pullulanase	27.5 ± 1.3
Xylanase	43.5 ± 2.1
Cellulase	32.0 ± 1.6
Pectinase	42.4 ± 1.7

**Notes.**

a Cassava pulp.

b soluble starch from potato were used as the substrates for assay.

### The complete genome of *C. manihotivorum* CT4^T^ and comparative genomics

**Figure 3:**
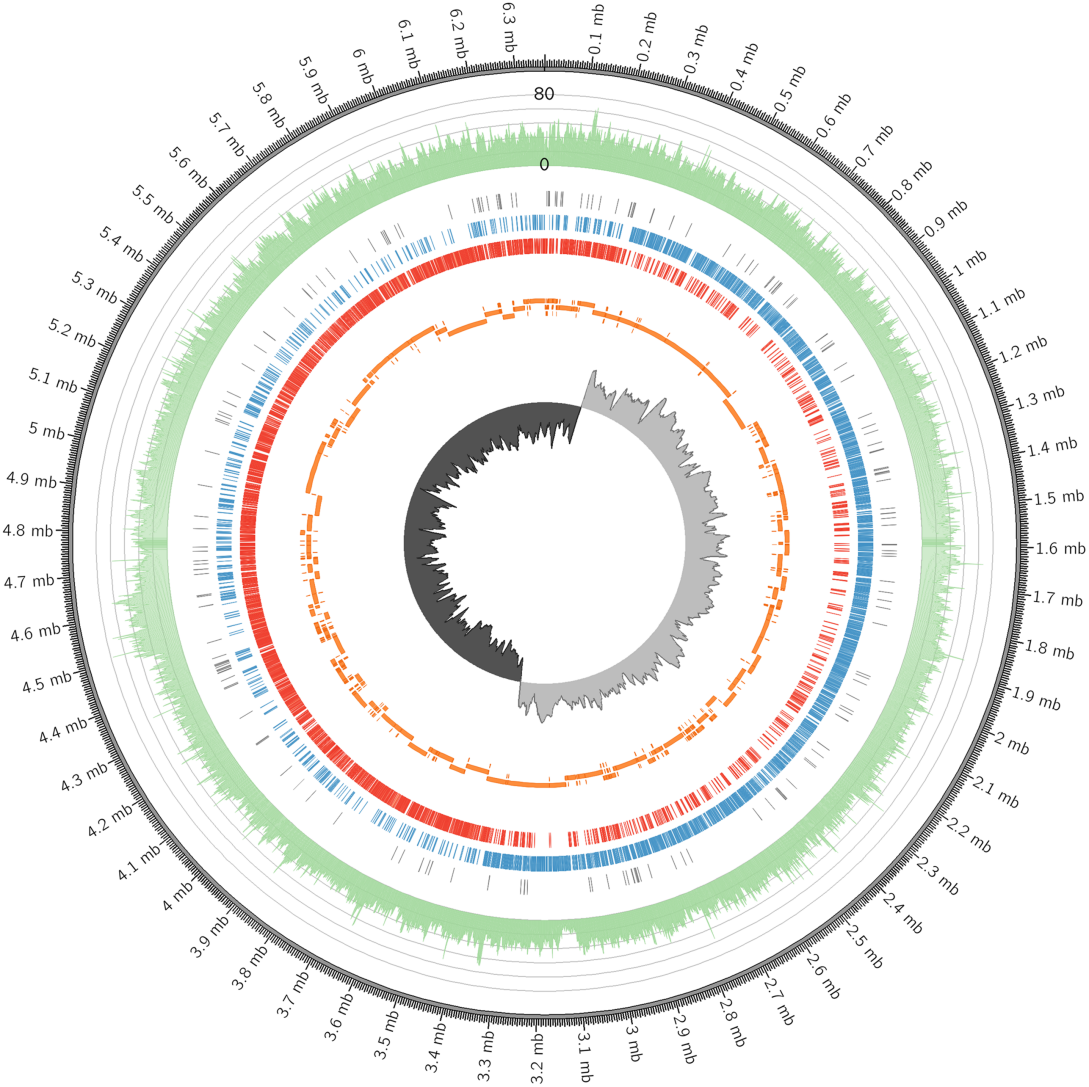
The circular genome map of *C. manihotivorum* CT4^T^.

In this study, the complete genome of *C. manihotivorum* CT4^T^, deposited in GenBank under the accession number CP025746, was described. A complete, gapless and circular genome assembly was generated, with a total size of 6,364,326 bases and a 40-fold coverage, as shown in Fig. 3 and Table 3. The origin of replication was determined based on GC skew analyses. The average G + C content was approximately 32 mol%, and plasmid was not detected. The DNA G + C content of strain CT4 (32 mol%), was within the range of 23–37% reported for the genus *Clostridium* (Lawson & Rainey, 2016). Genome annotation was performed using Prokka (Seemann, 2014) and Blast2GO (Conesa et al., 2005). The genome was predicted to have 5,664 protein-coding sequences (CDS), 42 rRNA sequences, 95 tRNA sequences, 1 tmRNA sequence and 153 misc_RNA sequences. Furthermore, NCBI Prokaryotic Genome Annotation Pipeline (PGAP) version 4.11 was also employed to annotate the genome, which provided slightly different result of 5,308 CDSs and 5,654 total genes (Table 3). Hereafter, 5,664 CDSs were used for further analysis, in which the details are available in Data S1. According to the comparison of the genomes between *C. manihotivorum* CT4^T^ and *C . amylolyticum* SW408^T^, the strain CT4^T^ has much larger genome size than the strain SW408^T^ about 2.1 Mb. Moreover, 5,664 CDSs were predicted in *C. manihotivorum* CT4^T^ whereas only 3,957 CDSs were reported in *C. amylolyticum* SW408^T^.

### Average amino acid identity and phylogenetic analysis

**Figure 4:**
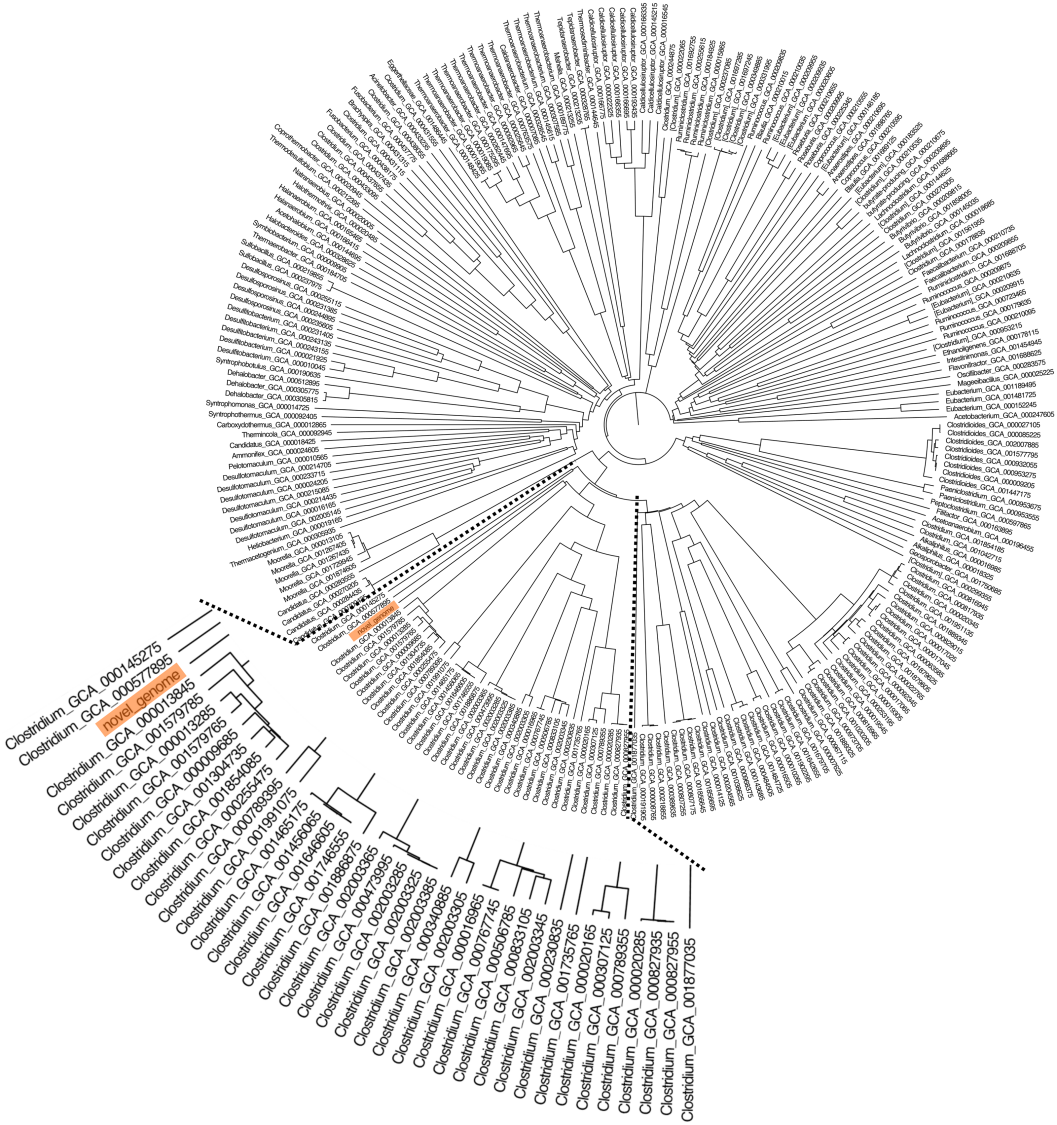
The AAI-based tree of *C. manihotivorum* CT4^T^ and related strains belonging to the 665 Clostridia class.

The whole-genome phylogeny of *C. manihotivorum* CT4^T^ was compared with a unique set of 665 Clostridia class genomes (Cabal et al., 2018), employing the average amino acid identity (AAI) analysis method. AAI has proven to have a better resolution power at the species level than 16S rRNA gene sequence-based comparison (Mahato et al., 2017). The derived phylogenetic tree based on the AAI analysis of all 666 genomes revealed several main clusters (Fig. 4). The strain CT4^T^ was clearly separated from the other Clostridia, in which a single branch was observed. The result aligned with the above physiological and biochemical characteristics that differ from other related type strains.

**Table 3:** The genome features of *C. manihotivorum* CT4^**T**^.

**Features**	**Values**	
	**In-house pipeline**	**PGAP pipeline**
Genome size (bp)	6,364,326	6,364,326
G + C content (mol%)	32	32
Total number of genes	5,941	5,654
Protein-coding sequences	5,664	5,308
rRNA genes	42	42
tRNA genes	95	95
tmRNA	1	1
misc_RNA	153	153

### Functional category of strain CT4^T^

Approximately, 75% (4,223 out of 5,664) of the protein-coding sequences in *C. manihotivorum* CT4^T^ were classified into COG functional categories (Table 4): replication, recombinant and repair (L: 548 protein-coding sequences); transcription (K: 381); carbohydrate transport and metabolism (G: 318); amino acid transport and metabolism (E: 276); cell wall/membrane/envelope biogenesis (M: 260) and translation, ribosomal structure and biogenesis (J: 197), based on Tatusov et al. (2000). The COG category (G: 318) comprised mainly protein-coding sequences that were involved in the degradation of starch and polysaccharides contained in lignocellulosic materials, and the transportation of the compounds (Tomazetto et al., 2016). These results suggest that *C. manihotivorum* CT4^T^ contains genes encoding glycoside hydrolases, related to starch, hemicellulose, cellulose and pectin degrading enzymes (Table 5).

**Table 4:** The Cluster of Orthologous Group (COG) functional categories of *C. manihotivorum* CT4^T^ genome.

**Code**	**Functional annotation**	**Number of genes**
B	Chromatin structure and dynamics	1
C	Energy production and conversion	172
D	Cell cycle control, cell division, chromosome partitioning	38
E	Amino acid transport and metabolism	276
F	Nucleotide transport and metabolism	82
G	Carbohydrate transport and metabolism	318
H	Coenzyme transport and metabolism	97
I	Lipid transport and metabolism	71
J	Translation, ribosomal structure and biogenesis	197
K	Transcription	381
L	Replication, recombination and repair	548
M	Cell wall/membrane/envelope biogenesis	260
N	Cell motility	56
O	Posttranslational modification, protein turnover and chaperones	109
P	Inorganic ion transport and metabolism	30
T	Signal transduction mechanisms	41
U	Intracellular trafficking, secretion and vesicular transport	153
R	General function prediction only	1,134
S	Function unknown	259
**Total**		**4,223**

**Table 5:** The genes encoding for amylolytic-, hemicellulolytic- and cellulolytic-enzymes in the genome of *C. manihotivorum*CT4^T^. The bold locus tag indicates the presence of CBM domains in their structure. The domains were identified using the conserved database domain (at NCBI), dbCAN and InterProScan.

**Enzymes**	**EC number**	**Locus tag**	**Domains organization**
**Amylolytic enzymes**			
*α*-Amylase	3.2.1.1	CT4_03811, CT4_04618, CT4_04619, CT4_04620, CT4_04873, **CT4_05358**, **CT4_01439**	**CT4_05358:** GH13–CBM20 **CT4_01439:** GH13–CBM53–CBM53
Oligo- *α*-1,6-glucosidase	3.2.1.10	CT4_03811, CT4_04618, CT4_04619, CT4_04620, CT4_04873	
*α*-Glucosidase	3.2.1.20	CT4_03811, CT4_04618, CT4_04619, CT4_04620, CT4_04873, CT4_01877, CT4_03509, CT4_00906, **CT4_04500**, **CT4_04692**, CT4_05353, CT4_05689	**CT4_04500:** CBM34–GH13 **CT4_04692:** CBM34–GH13
Amylo- *α*-1,6-glucosidase	3.2.1.33	CT4_04498	
Pullulanase	3.2.1.41	CT4_00906	
Glucan- *α*-1,6-glucosidase	3.2.1.70	CT4_03811, CT4_04618, CT4_04619, CT4_04620, CT4_04873	
**Hemicellulolytic enzymes**			
Endo-1,4-*β*-xylanase	3.2.1.8	CT4_03195, CT4_04894	
*α*-Galactosidase	3.2.1.22	CT4_04979, CT4_04272	
*β*-Galactosidase	3.2.1.23	CT4_00135, CT4_01004, CT4_01881, CT4_02219, CT4_05037, CT4_05052, CT4_05461, CT4_00379, CT4_01609, CT4_03387	
*β*-Glucuronidase	3.2.1.31	CT4_00135, CT4_01004, CT4_01881, CT4_02219, CT4_05037, CT4_05052, CT4_05461, CT4_04896	
*β*-Xylosidase	3.2.1.37	CT4_03273	
*α*-L-Arabinofuranosidase	3.2.1.55	CT4_03251, **CT4_03484**, **CT4_03690**, CT4_02877, CT4_03686	**CT4_03484:** CBM4–GH43 **CT4_03690:** CBM4–GH43
Endo-*β*-1,4-mannanase	3.2.1.78	**CT4_04971, CT4_05469**	**CT4_04971:** CBM6–CBM35–GH26 **CT4_05469:** CBM6–CBM35–GH26
Endo-*α*-1,5-L-arabinanase	3.2.1.99	CT4_01164, CT4_03483, CT4_03685, CT4_05022	
**Cellulolytic enzymes**			
Endo-*β*-1,4-glucanase	3.2.1.4	**CT4_03367**, CT4_01165 **CT4_00352, CT4_05071**	**CT4_03367:** GH9–CBM3–CBM3 **CT4_00352:** GH5–CBM46 **CT4_05071:** GH5–CBM46–CBM3
*β*-Glucosidase	3.2.1.21	CT4_00135, CT4_01004, CT4_01881, CT4_02219, CT4_05037, CT4_05052, CT4_05461, CT4_00940, CT4_01878, CT4_02623, CT4_03388, **CT4_03385**	**CT4_03385:** GH3–CBM6
Endo-*β*-1,6-glucanase	3.2.1.75	CT4_00944	
6-Phospho-*β*-glucosidase	3.2.1.86	CT4_00135, CT4_01004, CT4_01881, CT4_02219, CT4_05037, CT4_05052, CT4_05461, CT4_05778	
**Pectinolytic enzyme**			
Pectate lyase	4.2.2.2	CT4_00924	

### Identification of the genes encoding amylolytic-, hemicellulolytic-, cellulolytic- and pectinolytic-enzymes in *C. manihotivorum* CT4^T^

The genes encoding amylolytic-, hemicellulolytic-, cellulolytic- and pectinolytic-enzymes were detected in the genome of *C. manihotivorum* CT4^T^ (Table 5). Amylolytic enzymes were found in the complete genome of strain CT4^T^ including *α*-amylase, oligo-*α*-1,6-glucosidase, *α*-glucosidase, amylo-*α*-1,6-glucosidase, pullulanase and glucan-*α*-1,6-glucosidase that could be classified into endo-acting amylase, exo-acting amylase and debranching amylase. The *α*-amylase of *C. manihotivorum* CT4^T^ was predicted to contain starch binding domains (SBDs) of CBM20 (gene locus; CT4_05358) and CBM53 (CT4_01439) while *α*-glucosidase contained CBM34 (CT4_04500 and CT4_04692), as shown in Table 5. Moreover, the genes encoding hemicellulolytic- and cellulolytic-enzymes, such as endo-1,4-*β*-xylanase, *α*-galactosidase, *β*-galactosidase, *β*-glucuronidase, *β*-xylosidase, *α*-L-arabinofuranosidase, endo-*β*-1,4-mannanase, endo-*α*-1,5-L-arabinanase, endo-*β*-1,4-glucanase, *β*-glucosidase, endo-*β*-1,6-glucanase and 6-phospho-*β*-glucosidase, have been observed to be involved in the hydrolysis of hemicellulose and cellulose. In addition, the genome of *C. manihotivorum* CT4^T^ also harbors a gene (CT4_00924), which encodes a putative pectate lyase that can be hydrolyzed the internal *α*-1,4 linked D-galacturonic acid within the pectin.

As illustrated in Fig. 1, phylogenetic analyses showed that strain CT4^T^ forms a cluster with *C. polyendosporum* PS-1^T^. The latter cluster forms a sibling group with the *C. amylolyticum* SW408^T^, *C. putrefaciens* DSM 1291^T^ and *C. algidicarnis* NCFB 2931^T^ branch. However, only the strain SW408^T^ was released as a genome announcement. Thus, the *C. amylolyticum* SW408^T^ was chosen for comparative genomic exploration. Analysis of genes encoding carbohydrate-active enzymes in the genomes of strains CT4^T^ and SW408^T^ revealed differences in the distribution of genes (Table 6). *C. manihotivorum* CT4^T^ had a higher number of genes encoding amylolytic enzymes than that of *C. amylolyticum* SW408^T^. Moreover, strain CT4^T^ contained more genes encoding hemicellulolytic-, cellulolytic- and pectinolytic-enzymes than *C. amylolyticum* SW408^T^, except the debranching enzyme, *α*-galactosidase.

**Table 6:** The comparison of genes encoding for amylolytic-, hemicellulolytic- and cellulolytic-enzymes in *C. manihotivorum* CT4^**T**^ and *C. amylolyticum* SW408^**T**^.

**Enzymes**	**EC number**	**Strains**		
		**CT4**	**SW408**
**Amylolytic enzymes**			
*α*-Amylase	3.2.1.1	7	2	
Oligo-*α*-1,6-glucosidase	3.2.1.10	5	1	
*α*-Glucosidase	3.2.1.20	12	8	
Amylo-*α*-1,6-glucosidase	3.2.1.33	1	0	
Pullulanase	3.2.1.41	1	0	
Glucan-*α*-1,6-glucosidase	3.2.1.70	5	1	
	**Total**	**31**	**12**	
**Hemicellulolytic enzymes**			
Endo-1,4-*β*-xylanase	3.2.1.8	2	0	
*α*-Galactosidase	3.2.1.22	2	5	
*β*-Galactosidase	3.2.1.23	10	4	
*β*-Glucuronidase	3.2.1.31	8	3	
*β*-Xylosidase	3.2.1.37	1	0	
*α*-L-Arabinofuranosidase	3.2.1.55	5	0	
Endo- *β*-1,4-mannanase	3.2.1.78	2	0	
Endo-*α*-1,5-L-arabinanase	3.2.1.99	4	0	
	**Total**	**34**	**12**	
**Cellulolytic enzymes**			
Endo-*β*-1,4-glucanase	3.2.1.4	4	0	
*β*-Glucosidase	3.2.1.21	12	3	
Endo-*β*-1,6-glucanase	3.2.1.75	1	0	
6-Phospho-*β*-glucosidase	3.2.1.86	8	4	
	**Total**	**25**	**7**	
**Pectinolytic enzyme**			
Pectate lyase	4.2.2.2	1	0	
	**Total**	**1**	**0**	

## Discussion

As we know, cassava pulp generated in large amounts, as industrial waste during cassava processing is rich in starch and fiber (Norrapoke et al., 2018). Thus, it can be used as a renewable material to produce high value-added products (FitzPatrick et al., 2010). Mostly, bacterial species of the genus *Clostridium* are known as good degraders of lignocellulosic materials (Doi & Kosugi, 2004). However, not much is known regarding amylase, hemicellulase and cellulase-producing species that are capable of efficient cassava pulp degradation. Among the species isolated from soil samples collected from cassava pulp landfill using the Hungate roll-tube technique, strain CT4^T^ was most effective in degrading cassava pulp. The roll-tube procedure has previously been used to isolate single colonies and pure cultures of bacteria, including *Clostridium thermocellum* S14 (Tachaapaikoon et al., 2012) and *C . amylolyticum* SW408^T^ (Song & Dong, 2008). Subsequently, strain CT4^T^ was identified using the 16S rRNA gene sequencing analysis. According to 16S rRNA gene sequence analysis, strain CT4^T^ was phylogenetically related to members of the genus *Clostridium* (90–95% sequence similarity), with the highest degree of sequence similarity to *C. polyendosporum* PS-1^T^ (95%) and follow by *C . amylolyticum* SW408^T^ (94%). These values are at the level suggested to allocate the strain to a novel species of genus *Clostridium* (Yarza et al., 2008). Moreover, AAI and phylogenetic analysis of the strain CT4^T^ suggested that the newly isolated strain CT4^T^ should be classified as a novel species of the genus *Clostridium*, known as *C. manihotivorum* CT4^T^.

Although *C. polyendosporum* PS-1^T^ could degrade starch, its activity is not known (Duda et al., 1987). Remarkably, *C. polyendosporum* PS-1^T^ and *C. manihotivorum* CT4^T^ have different capacities for endogenous spore formation. While *C. polyendosporum* PS-1^T^ has the ability to form several endospores in one cell (some cells may produce up to seven), cells of the strain CT4^T^ contained a single endospore (Table 1). Likewise, *C. amylolyticum* SW408^T^, a mesophilic anaerobic amylolytic bacterium (and a close relative of strain CT4^T^), isolated from an H_2_-producing up-flow anaerobic sludge blanket reactor utilizes several kinds of mono- and di-saccharides and simultaneously hydrolyzes and ferments starch (Song & Dong, 2008). Nonetheless, there are no reports precisely in relation to cassava pulp degradation in this genus. The degradation of cassava pulp by strain CT4^T^ was also compared with that of *C. polyendosporum* PS-1^T^ and *C. amylolyticum* SW408^T^, which are the closest related species. They were inoculated into BM7 containing 1% (w/v) cassava pulp at 37 °C, pH 7.0 for 5 days. *C. manihotivorum* CT4^T^ grew rapidly, while both strains showed a small amount of growth on cassava pulp. After cultivation, the residue weights of *C. manihotivorum* CT4^T^, *C. polyendosporum* PS-1^T^ and *C. amylolyticum* SW408^T^ were decreased by 60.8% (w/v), 0.6% (w/v) and 0.4% (w/v), respectively, and compared with the initial dry weight of cassava pulp. Cassava pulp compositions after digested by *C. manihotivorum* CT4^T^, *C. polyendosporum* PS-1^T^ and *C. amylolyticum* SW408^T^ were analyzed (Fig. S2). *C. manihotivorum* CT4^T^ showed a high degradation ability for starch, which was 99% starch removal. Moreover, the strain CT4^T^ revealed not only efficient starch degradation but also cellulose, hemicellulose and pectin. By contrast, *C. polyendosporum* PS-1^T^ and *C. amylolyticum* SW408^T^ showed ineffective cassava pulp degradation. The starch, cellulose, and hemicellulose contents of the residues were little decreased. The result indicated that *C. manihotivorum* CT4^T^ might have better cassava pulp degradation ability than *C. polyendosporum* PS-1^T^ and *C. amylolyticum* SW408^T^. The results indicated that the *C. manihotivorum* CT4^T^ showed greater cassava pulp degradation than the other closely related species. The significantly different degradation of cassava pulp by the crude enzyme from *C. manihotivorum* CT4^T^, from the other members of *Clostridium*, was possibly caused by many factors such as: (1) synergistic interactions among amylolytic-, hemicellulolytic- and cellulolytic-enzymes; (2) the enzymes containing non-catalytic binding domains that linked with catalytic domains known as carbohydrate-binding modules (CBMs); and (3) the weak binding of the enzymes to lignin. Various hemicellulolytic-enzymes including endo-*β*-1,4-xylanase, *β*-xylosidase and endo-*β*-1,4-mannanase were broken down xylan and mannan, the main hemicellulose in cassava pulp that covers the cellulose. Removal of xylan and mannan could help to increase the accessibility of cellulolytic-enzymes (such as endo- *β*-1,4-glucanase and *β*-glucosidase) for disruption of cellulose. Synergism between hemicellulolytic- and cellulolytic-enzymes led to enhanced release of the entrapped starch granules from cassava pulp. Consequently, the entrapped starch granules became more available for amylolytic-enzyme which were then effectively hydrolyzed to oligosaccharides and monosaccharides by endo-acting *α*-amylase, exo-acting *α*-glucosidase, and debranching enzyme pullulanase. Therefore, the synergism hemicellulolytic- and cellulolytic-enzymes acted cooperatively on decomposition of the hemicellulose-cellulose matrix, leading to increased accessibility of the amylolytic enzymes to the exposed starch granule located within the cassava pulp (Bunterngsook et al., 2017; Poonsrisawat et al., 2017), while CBMs have been reported to assist hydrolysis of insoluble substances by bringing the catalytic domain in close proximity to its substrate (Hervé et al., 2010). Moreover, the cassava pulp degrading enzyme of *C. manihotivorum* CT4^T^ may be active and low binding to lignin in cassava pulp. Teeravivattanakit et al. (Teeravivattanakit et al., 2017) reported that because the bacterial multifunctional enzyme PcAxy43A from *Paenibacillus curdlanolyticus* B-6 was a weak lignin-binding enzyme, this enzyme was capable of converting xylan contained in agricultural residues to xylose in one step without chemical pretreatment to remove lignin. Therefore, a weak lignin-binding enzyme is a potential factor for obtaining enzymes suitable for the hydrolysis of lignocellulosic materials (Berlin et al., 2006). Although some *Clostridium* spp. such as *C. amylolyticum* SW408^T^ (Song & Dong, 2008), *C. thermosulfurigenes* H12-1 (Saha, Shen & Zeikus, 1987) and *C. butyricum* T-7 (Tanaka et al., 1987) have the ability to hydrolyze soluble starch or raw starch by producing *α*-amylase and *β*-amylase. However, these three strains do not produce pullulanase, xylanase or cellulase and thus, unlike *C. manihotivorum* CT4^T^, lack the properties of cassava pulp degrading enzymes.

To further explore whether *C. manihotivorum* CT4^T^ could be used to degrade cassava pulp, we analyzed its whole genome for the presence of enzymes involved in cassava pulp degradation. It found that the genome contains various genes encoding amylolytic-, hemicellulolytic- and cellulolytic-enzymes which possess different CBM domains. Those CBM families help in substrate recognition and binding, and thus increase the catalytic activity on insoluble substrates such as CBM20, CBM34 and CBM53 have been reported to act in the degradation of raw starch granules by enabling the enzyme to interact with the starch granules and also disrupt the surface of the starch structure (Machovič & Janeček, 2006; Lombard et al., 2014). Furthermore, hemicellulases and cellulases, including the exo-, endo-types and side-chain acting enzymes, are involved in the hydrolysis of hemicellulose and cellulose contained in lignocellulosic materials (Linares-Pastén, Andersson & Karlsson, 2014). The genome annotation of *C. manihotivorum* CT4^T^ revealed the presence of gene products of hemicellulases featuring CBMs that have the ability to interact with insoluble substances and support catalytic domains to hydrolyze their substrates (Shallom & Shoham, 2003). For example, the *α*-L-arabinofuranosidase and endo-*β*-1,4-mannanase of *C. manihotivorum* CT4^T^ were predicted to contain CBM4 (gene loci; CT4_03484 and CT4_03690), CBM6 and CBM35 (CT4_04971 and CT4_05469) which have been reported to have a binding function to insoluble xylan (Munir et al., 2014). However, endo-1,4- *β*-xylanases (gene loci; CT4_03195 and CT4_04894), the main enzymes to attack the xylan backbone of strain CT4^T^ could not find the CBM. To explain how those xylanases were able to degrade xylan in cassava pulp, the enzymes might have other substrate-binding regions which are located at a certain distance from the active site and are called secondary xylan-binding sites (SXS), which function similarly to the CBM (Jommuengbout et al., 2009). Based on the amino acid sequence alignment of endo-1,4-*β*-xylanase (CT4_04894) with an endo-1,4-*β*-xylanase in the glycoside hydrolase family 10 (Xyn10) from *Penicillium simplicissimum*, which is capable of binding to insoluble xylan via the SXS, it was found that the residues E60, N61, K64, H97, W101, N142, E143, Y187, Q218, H220, E250, W283 and W291 of an endo-1,4-*β*-xylanase from strain CT4^T^ (CT4_04894) were conserved with the SXS of Xyn10 from *P. simplicissimum* (Schmidt, Gübitz & Kratky, 1999). Besides, cellulolytic enzymes such as endo-*β*-1,4-glucanase and *β*-glucosidase in *C. manihotivorum* CT4^T^ also contained CBM3 (gene locus; CT4_03367), CBM6 (CT4_03385), CBM3 and/or CBM46 (CT4_05071 and CT4_00352), which are known to bind and support catalytic domains to hydrolyze crystalline and amorphous celluloses (Cho et al., 2008; Guillén, Sánchez & Rodríguez-Sanoja, 2010). The results strongly indicated that *C. manihotivorum* CT4^T^ possesses a set of genes encoding a complete system of amylolytic-, hemicellulolytic- and cellulolytic-enzymes, indicating that *C. manihotivorum* CT4^T^ is a good candidate for degrading cassava pulp.

## Conclusions

In this work, we have highlighted the cassava pulp-degrading enzyme of the isolated strain CT4^T^. It is a new species of the genus *Clostridium* that possesses specialized ability to degrade cassava pulp, a property that is occasionally found in this genus. The AAI constructed from *C. manihotivorum* CT4^T^ revealed differences in the evolutionary relationships among the other *Clostridium* species. A complete genome sequence studied by Illumina and Oxford Nanopore Technology revealed that *C. manihotivorum* CT4^T^ possesses a set of genes encoding the enzymes for the decomposition of an industrial starch-rich by-product, cassava pulp. In addition, *C. manihotivorum* CT4^T^ contained a total of 91 genes encoding amylolytic-, hemicellulolytic-, cellulolytic- and pectinolytic-enzymes. Comparative analyses of the *C. manihotivorum* CT4^T^ with the genome of *C. amylolyticum* SW408^T^ revealed that strain CT4^T^ had a high proportion and diversity of amylolytic-, hemicellulolytic-, cellulolytic- and pectinolytic-enzymes. The results suggest that *C. manihotivorum* CT4^T^ is a promising microbe for saccharification of cassava pulp into useful value-added products.

### Description of *Clostridium manihotivorum* sp. nov.

*Clostridium manihotivorum* sp. nov. (ma.ni.ho.ti.vo’rum. N.L. n. *manihot*, a botanical genus name (cassava); L. v. *voro*, to eat, devour; N.L. neut. adj. *manihotivorum*, devouring cassava).

Cells are Gram-positive, anaerobic, rods, single-endospore forming, non-motile and non-flagellate. Cells are 4.0–8.0 µm long and 0.5–1.5 µm wide. Colonies are 0.5–1.0 mm in diameter after incubation on BM7 agar supplemented with cassava pulp (1%, w/v) at 37 °C for 5 days. Cell growth is observed at 25−45 °C (optimum, 37 °C) and at pH 5.5–7.5 (optimum, 7.0). The strain curdles milk, but is negative for catalase, H_2_S, and indole. Utilizes D-glucose, D-xylose, D-galactose, D-fructose, D-mannose, D-arabinose, D-rhamnose, D-trehalose, D-raffinose, sucrose, lactose, maltose, mannitol, cellobiose, soluble starch, xylan, cellulose and Avicel^®^. The end products of glucose fermentation are acetate, butyrate, ethanol, propionate, CO_2_ and H_2_. The diagnostic amino acid in their cell wall is *LL*-diaminopimelic acid (*LL*-DAP). The major fatty acids are C _16:0_, C _14:0_, anteiso-C _15:0_, summed feature 1 (C _13:0_-3OH and/or C _15:1_isoH), C _19:0_cyclo *ω*8c and C _17:0_2-OH. The major polar lipids present are phosphatidylethanolamine (PE) and phosphatidylglycerol (PG). The genome size of the type strain is around 6.3 Mb and the genomic DNA G + C content 32 mol%. The type strain, CT4^T^ (=TBRC 11758^T^ = NBRC 114534^T^), was isolated from soil collected from a cassava pulp landfill located at Chonburi province, Thailand.

##  Supplemental Information

10.7717/peerj.10343/supp-1 Figure S1 The two-dimensional thin-layer chromatograms of the polar lipids from *C. manihotivorum* CT4^*T*^ detected with the following reagents: phosphomolybdic acid (A), Dittmer and Lester reagent (B), ninhydrin reagent (C) and Dragendorff’s reagent (D). PE, phosphatidylethanolamine; PG, phosphatidylglycerol; PC, phosphatidylcholine; AL1-AL3, unidentified aminolipids; PL1-PL3, unidentified phospholipids.

10.7717/peerj.10343/supp-2 Data S1 Protein-coding sequences of *C. manihotivorum* CT4^*T*^

10.7717/peerj.10343/supp-3 Table S1 The cellular fatty acid compositions of *C. manihotivorum* CT4^*T*^

10.7717/peerj.10343/supp-4 Table S2 The raw data set of enzyme activities produced by C. manihotivorum CT4^*T*^

10.7717/peerj.10343/supp-5 Figure S2 Biodegradation ability for cassava pulp by *C. manihotivorum* CT4^*T*^ , *C. polyendosporum* PS-1^*T*^ and *C. amylolyticum* SW408^*T*^ The chemical compositions of residual cassava pulp relative to the original weight is shown after cultured with CT4^*T*^ , PS-1^*T*^ and SW408^*T*^ for 5 days.

## Additional Information and Declarations

### Data Availability

The following information was supplied regarding data availability: The complete genome of *C. manihotivorum* CT4^T^ is available at GenBank: CP025746.

### New Species Registration

The following information was supplied regarding the registration of a newly described species: The registration of *Clostridium manihotivorum* CT4 was deposited as a type strain in the Thailand Bioresource Research Center (TBRC): TBRC 11758^T^; and at NITE Biological Resource Center (NBRC), Japan: NBRC 114534^T^.
