# Supplementary figures and images for: Clostridium manihotivorum sp. nov., a novel mesophilic anaerobic bacterium that produces cassava pulp-degrading enzymes
Source: PeerJ. 2020 Nov 16;8:e10343. doi: 10.7717/peerj.10343 (PMC7676355; doi:10.7717/peerj.10343)

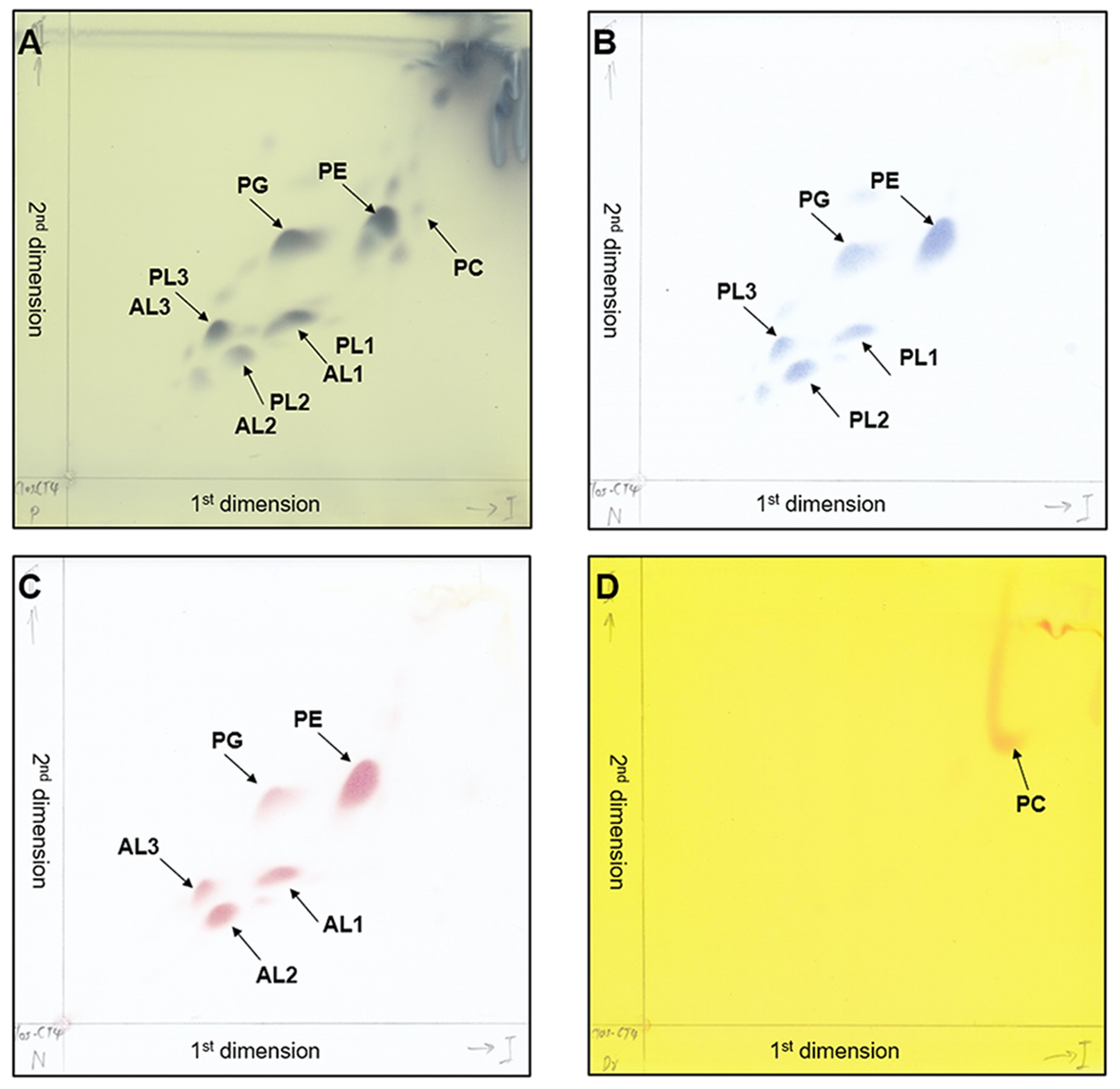

Supplement: Figure S1 — phosphomolybdic acid (A), Dittmer and Lester reagent (B), ninhydrin reagent (C) and Dragendorff’s reagent (D). PE, phosphatidylethanolamine; PG, phosphatidylglycerol; PC, phosphatidylcholine; AL1-AL3, unidentified aminolipids; PL1-PL3, unidentified phospholipids. [file peerj-08-10343-s001.png]
