# Supplementary material for: Clostridium manihotivorum sp. nov., a novel mesophilic anaerobic bacterium that produces cassava pulp-degrading enzymes
Source: PeerJ. 2020 Nov 16;8:e10343. doi: 10.7717/peerj.10343 (PMC7676355; doi:10.7717/peerj.10343)
Supplement: Table S1 [file peerj-08-10343-s003.docx]

**Supplemental Table S1: The cellular fatty acid compositions of *C. manihotivorum* CT4^T^.** The values are percentages of the total fatty acids. The fatty acids amounting to <1.0% of the total fatty acids are not shown

| **Fatty acids** | **Strain CT4** |
| --- | --- |
| C_12:0_ | 2.2 |
| C_14:0_ | 15.0 |
| C_16:0_ | 37.4 |
| iso-C_16:0_ | 1.4 |
| anteiso-C_15:0_ | 5.5 |
| anteiso-C_17:0_ | 1.0 |
| C_16:1_ω9c | 2.6 |
| C_18:1_ω5c | 1.3 |
| C_15:0_ 2-OH | 2.0 |
| C_17:0_ 2-OH | 4.0 |
| C_16:0_ N alcohol | 2.3 |
| C_17:0_ cyclo | 1.4 |
| C_19:0_ cyclo ω8c | 4.2 |
| Summed feature 1 | 4.5 |
| Summed feature 3 | 3.3 |
| Summed feature 8 | 3.4 |

Summed features represent groups of two fatty acids which could not be separated by GLC and the MIDI system, such as summed feature 1 containing C_13:0_-3OH and/or C_15:1_ isoH, summed feature 3 containing C_16:1_ω6c and/or C_16:1_ω7c, and summed feature 8 containing C_18:1_ω6c and/or C_18:1_ω7c.
